# Correlated Fluctuations of Structural Indicators Close to the Liquid–Liquid Transition in Supercooled Water

**DOI:** 10.1021/acs.jpcb.2c07169

**Published:** 2022-12-20

**Authors:** Riccardo Foffi, Francesco Sciortino

**Affiliations:** † Institute for Environmental Engineering, Department of Civil, Environmental and Geomatic Engineering, ETH Zürich, 8093Zürich, Switzerland; ‡ Dipartimento di Fisica, Sapienza Università di Roma, Piazzale Aldo Moro 5, I-00185Rome, Italy

## Abstract

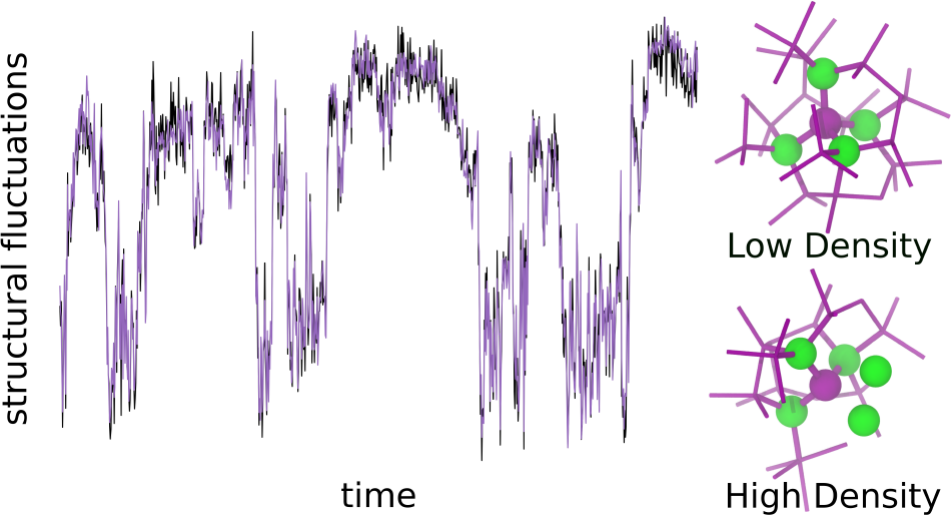

Multiple numerical studies have unambiguously shown the existence of a liquid–liquid critical point in supercooled states for different numerical models of water, and various structural indicators have been put forward to describe the transformation associated with this phase transition. Here we analyze numerical simulations of near-critical supercooled water to compare the behavior of several of such indicators with critical density fluctuations. We show that close to the critical point most indicators are strongly correlated to density, and some of them even display identical distributions of fluctuations. These indicators probe the exact same free energy landscape, therefore providing a thermodynamic description of critical supercooled water which is identical to that provided by the density order parameter. This implies that close to the critical point, there is a tight coupling between many, only apparently distinct, structural degrees of freedom.

## Introduction

Water has an intriguing thermodynamic behavior, which originates from its ability to alter its local structure in response to changes in pressure and temperature. The tetrahedral geometries characteristic of crystalline ice persist, locally, also in the liquid state, but depending on the external conditions, the hydrogen bond (HB) network of water can undergo drastic rearrangements, leading to significant distortions in the local environment.^1,2^ The thermodynamic anomalies of liquid water are therefore a direct result of the peculiar properties of the HB network, where local rearrangements are accompanied by an anticorrelation between local density and local energy, in stark contrast to the behavior of normal liquids.^3^

One of the most striking consequences is polyamorphism, the existence of multiple distinct amorphous phases for a single-component substance.^4,5^ In particular, two of these, the low-density amorphous (LDA) and high-density amorphous (HDA) phases, appear to be separated by a first-order transition.^6−8^ On the basis of numerical simulations, Poole et al.^9^ advanced the suggestive hypothesis that the LDA-HDA first-order transition could be the kinetically arrested manifestation of a liquid–liquid transition (LLT), occurring at deeply supercooled conditions and involving two distinct, metastable liquid states, a low- and a high-density liquid (LDL and HDL). This LLT line would terminate at a liquid–liquid critical point (LLCP). Despite many controversies,^10,11^ cutting-edge experimental efforts are now entering in the no-mans land,^12−16^ providing increasingly stronger hints at the possibility that the LLT actually exists in real water.^14^ In many numerical models of water, however, a LLT has been predicted to exist,^9,17−21^ including models based on neural network potentials^22^ and models including quantum nuclear effects.^23^ In other models, the existence of a liquid–liquid critical point has been rigorously proven.^24−30^ In silico, a liquid–liquid critical point has also been clearly identified in colloidal models mimicking, at the nano- and micron-scale, the tetrahedral binding of water.^31−34^

Insight into the properties of water can be gained through the use of two-state models,^35−41^ where (with some variations and subtleties) water is seen as a binary mixture of two competing and continuously interconverting local structures. There is, in particular, a tradition of classifying the molecular components of the liquid in terms of “structural indicators”, quantities that assign a scalar value to each molecule on the basis of local structural (or energetic) properties,^36,42−49^ allowing a distinction between structures that appear more LDL-like or HDL-like. More recently, further structural insight is also being provided by explicitly analyzing the topological properties of the HB network^50−52^ and other graph-theoretical approaches.^53^ A few studies tried to perform a comparison of certain aspects of structural indicators,^54,55^ but unfortunately not in the critical region of supercooled water.

One of the most prominent signatures of proximity to a critical point is the onset of critical density fluctuations, whose distribution is tightly related to the free energy landscape of the system.^56^ Performing numerical simulations of water close to the LLCP, these fluctuations can be probed, highlighting continuous crossovers between the two liquid states.^29^ It is interesting, in these conditions, to assess what other physical properties of the system are affected by the critical phenomenon, and to what extent these structural indicators are correlated to the density.

In this work we analyze tenths-of-μs-long molecular dynamics trajectories to explore how the presence of critical density fluctuations in supercooled water correlates to the behavior of structural indicators. We find that most commonly used structural indicators display a near-perfect correlation with the density, implying the existence of a tight coupling between the structural properties to which they are sensitive. Moreover, we find that some of the indicators show identical distributions of fluctuations close to the LLCP, leading to equivalent descriptions of the free energy landscape: density or any single one of these indicators are sufficient to completely (and identically) describe the thermodynamics of the LLT. Away from the LLCP, as the standard component of the free energy overcomes the critical one, the coupling between the different structural indicators is weakened and a one-to-one relationship to the density cannot be established anymore for most of them.

## Methods

Molecular dynamics simulations of TIP4P/Ice water^57^ were performed in the NPT ensemble using GROMACS 5.1.4^58^ in single precision. Integration of the equations of motion was performed with a leapfrog integrator with time step 2 fs, temperature coupling was controlled by a Nosé–Hoover thermostat^59^ with characteristic time 8 ps, and pressure coupling was controlled by an isotropic Parrinello–Rahman barostat^60^ with characteristic time 18 ps. Molecular constraints were implemented by a sixth-order LINCS algorithm.^61^ A cutoff distance 0.9 nm was selected for van der Waals forces and electrostatic interactions were evaluated with a fourth-order particle-mesh Ewald method,^62^ with a real-space cutoff of 0.9 nm. The main analysis was performed in the inherent structures^63^ (unless specified otherwise), obtained by minimization of the potential energy via the steepest descent algorithm (STEEP) in GROMACS, in double precision, with a force tolerance of 1 J mol^–1^ nm^–1^ and a maximum step size of 5 × 10^–4^ nm. Some of the analyses were also based on real dynamics trajectories.

70 μs long simulations at 188 K, 1675 bar, close to the critical point of TIP4P/Ice (estimated at 188.6 K and 1725 bar^29^), probing the critical density fluctuations, were performed with a small system of 300 molecules, sampling configurations at intervals of 40 ns. Other simulations, used to explore the behavior of structural indicators along isobars, were performed with 1000 molecules. In this case, the simulated time was longer than 40 μs at the lowest temperatures and configurations were sampled at intervals of 80 ns.

The presence of a H-bond between two water molecules was assessed following the definition by Luzar and Chandler;^64^ this information is needed to evaluate some of the structural indicators. According to this definition, two H_2_O molecules are H-bonded if their O···O distance is less than 3.5 Å and their HÔO angle (the minimal angle between the intramolecular OH bond and the intermolecular O···O line) is less than 30°. It was shown in refs (51) and (52) that this geometric definition is highly accurate over a wide range of conditions in supercooled TIP4P/Ice water (including conditions investigated herein), especially if performed on the inherent structures, in which thermal fluctuations have been removed.

## Structural Indicators

A structural indicator  assigns a scalar value to each molecule depending on its local environment, a value that can be interpreted as the “level of order” associated with the local structure, where the loose definition of order indicates, in analogy with the ice structure, the propensity to form open, tetrahedrally coordinated, local structures. These open tetrahedral local arrangements are favored at low temperature and low densities, and hence, in broad terms, density is negatively correlated with local order. Some of the indicators are positively correlated with order (i.e., they assign high values to highly ordered configurations); others have a negative correlation. For convenience, in the following analysis we will deliberately multiply all studied indicators by a factor  so that after the multiplication all of them are positively correlated with order: low values of  represent disordered structures (more HDL-like), and high values of  indicate ordered structures (LDL-like).

In this article we compare density and a comprehensive set of structural indicators, listed below.

- *q*_4_(44) is a measure of angular ordering, representing the level of tetrahedrality of the local structure around a selected molecule in terms of the angular arrangement of its four closest neighbors.
- *d*_5_(36) is the (oxygen–oxygen) distance of a molecule from its fifth-nearest neighbor; it has been suggested that *d*_5_ generally overestimates the level of order in the local structures.^39^
- LSI (Local Structure Index)^45^ is a radial measure of the variance of intermolecular distances between successive neighbors.
- ζ^46^ is the first parameter to explicitly account for H-bonding; it measures the distance between the nearest non-H-bonded and farthest H-bonded molecule, providing a measure of shell interpenetration. A particularly convenient property of ζ is the possibility to represent its distribution as the superposition of two Gaussian curves.
- *V*_4_(48) is the only energy-based descriptor in the set: for each molecule, all its pairwise interaction energies are evaluated and ranked based on their strength; *V*_4_ is the energy associated with the fourth-strongest interaction. *V*_4_ is expected to correspond to the typical linear HB energy in a local tetrahedral configuration, becoming weaker (less negative) when structural distortions are introduced. It correlates positively with density.
- NTC (Node Total Communicability)^53^ is a recently introduced graph-theoretical metric of the cumulative connectivity of each water molecule, highlighting the contribution of intermediate/long-range effects; it was found to be particularly sensitive to variations in high-density structures. High values of NTC represent high connectivity and higher values of density. It must be noted that links in the network are defined on the basis of a simple geometric oxygen–oxygen distance criterion, so the resulting connectivity pattern does not coincide with the HB network.
- Another recent topological indicator is the centerline helicity , a measure of the “degree of entangledness” in the HB network based on the identification of knots and ring structures.^34^
- Finally, we also present data based on a novel indicator, Ψ, introduced here for the first time. Recent studies^51,52^ have shown that molecules at chemical distance *D* = 4 (i.e., pairs of molecules separated by 4 links along the HB network) are characterized by typical distances that are very different between the LDL and HDL phases. In the LDL phase, these molecules are typically at large distances *r* ≈ 6 Å. In the HDL, instead, there is a significant probability of detecting these chemically distant pairs of molecules at close distances in real space, constituting interstitial pairs (*r* ≈ 3.5 Å). The value of Ψ of a molecule *m* is defined as the minimal real-space distance between molecules at chemical distance *D* = 4 from *m*. Further details on the definition of Ψ are reported in the Supporting Information, and a simple Julia script for the evaluation of Ψ has been made available via GitHub.^65^

All these indicators quantify local properties of single molecules, associating a value  to each molecule *m*. To associate a value to each configuration we can evaluate a system-level (or global) indicator by averaging over all molecules (*N*) in the sample: . The time sequence  of each global indicator, a measure of the time evolution of the average structural properties, can be correlated to density and to any other distinct structural indicator.

## Results and Discussion

**Figure 1:**
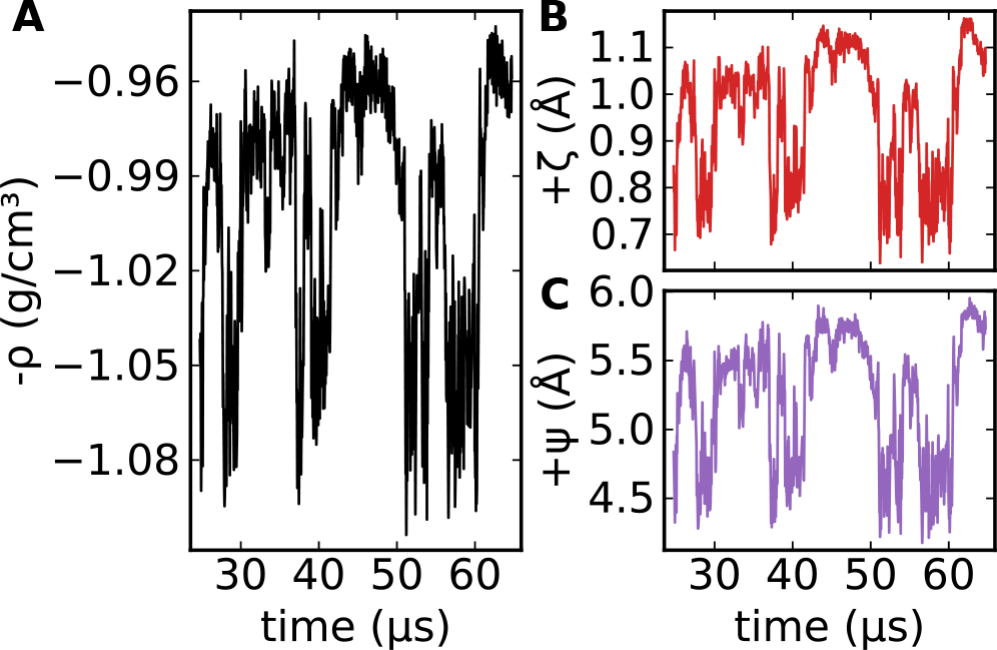
Time dependence of the (negative of the) density (A) and system-averaged structural indicators (B,C) from a constant-pressure simulation of TIP4P/Ice simulations at *T* = 188 K, *P* = 1675 bar (a state point close to the liquid–liquid critical point), and *N* = 300 molecules. The figure shows wide fluctuations between two distinct average values over μs time scales.

Figure 1A displays critical density fluctuations over a time scale of several μs observed in a constant-pressure molecular dynamics simulation of TIP4P/Ice water at *T* = 188 K, *P* = 1675 bar, a state point close to the model’s critical point (188.6 K and 1725 bar).^29^ In this condition, the density oscillates between values typical of the low- and the high-density liquid. Also the time evolution of structural indicators is characterized by wide fluctuations around two distinct average values, with frequent flips between the two, as shown in Figure 1B,C for +ζ and +Ψ, revealing that these indicators can detect the critical fluctuations; the same is true for all the other indicators considered here.

**Figure 2:**
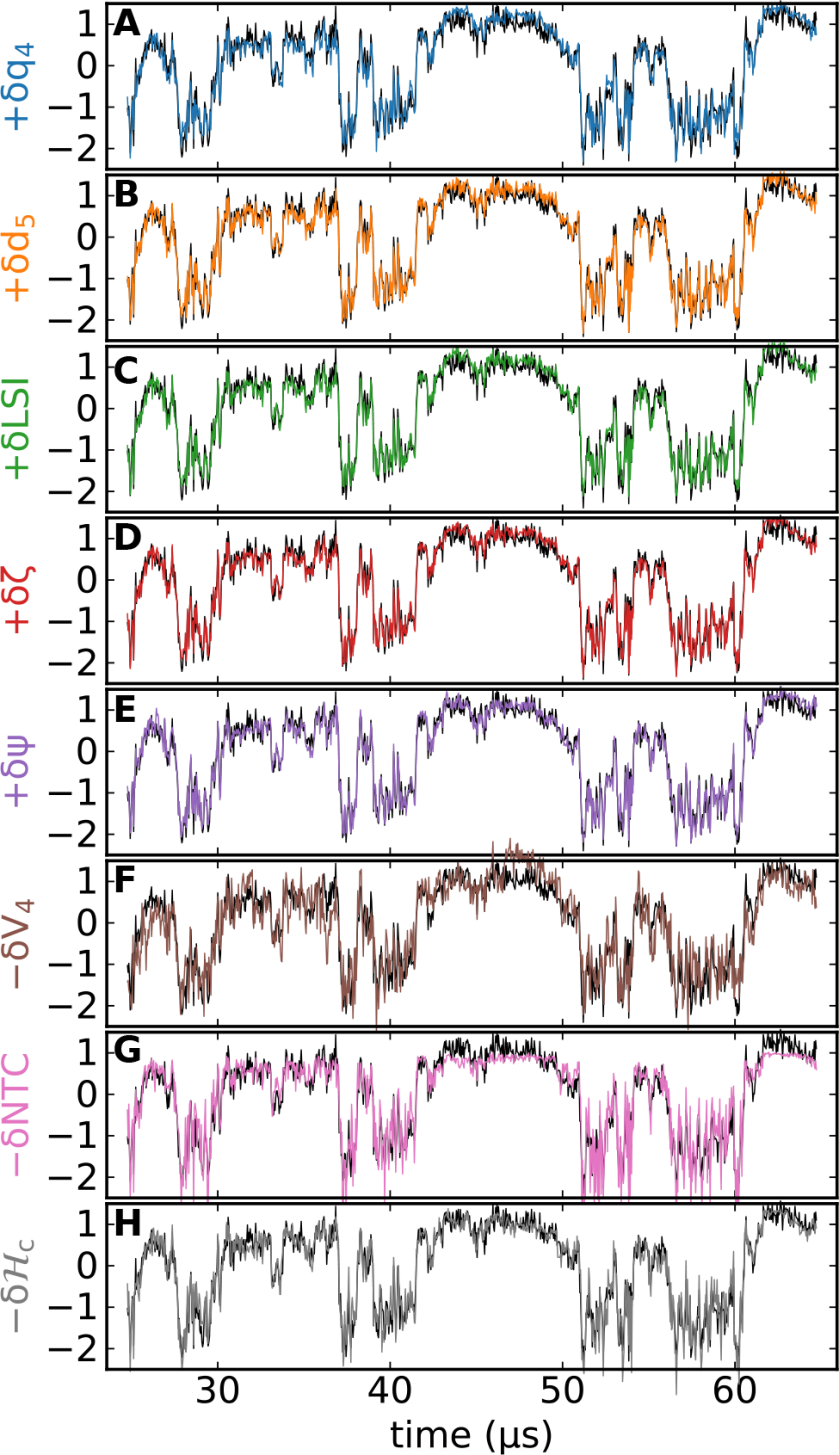
Critical density fluctuations (−*δρ*, black) compared to fluctuations of the structural indicators (color-coded): all indicators correlate strongly with density fluctuations in proximity of the critical point in TIP4P/Ice. Data from TIP4P/Ice simulations at *T* = 188 K, *P* = 1675 bar, and *N* = 300 molecules.

To quantify the ability of the investigated structural indicators to identify critical fluctuations (as detected by the oscillation in the system density) we define the fluctuation of a global indicator  as
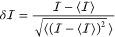
1where ⟨ · ⟩ denotes a time average. The resulting quantities  are dimensionless, all with zero mean and unit variance, allowing a direct comparison between the behavior of distinct structural indicators and density fluctuations. Figure 2 shows the time dependence of  for all investigated indicators, each of them superimposed with the density fluctuations (−*δρ*). In proximity of the LLCP, all the structural indicators we considered show a near-perfect correlation with the critical density fluctuations and between themselves.

**Figure 3:**
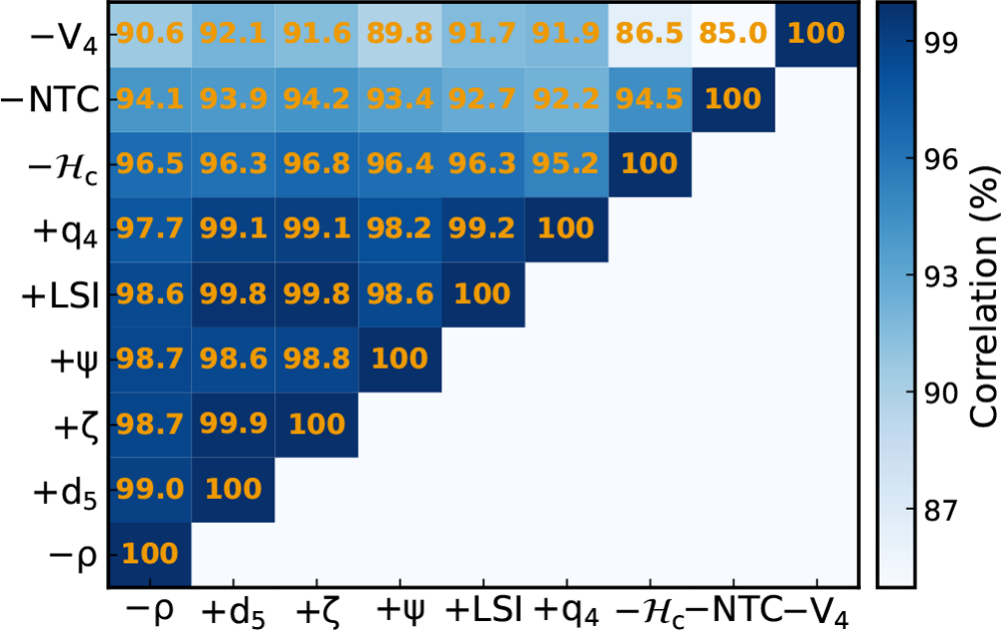
Pearson correlation coefficients between density and structural indicators and between pairs of structural indicators. Indicators are sorted, left to right and bottom to top, by their correlation with density. Data from TIP4P/Ice simulations at *T* = 188 K, *P* = 1675 bar, and *N* = 300 molecules.

The correlation between any two quantities  and  is quantified by the Pearson correlation coefficient
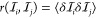
2where ⟨ · ⟩ is a time average. The resulting correlation coefficients (between density and structural indicators and between pairs of structural indicators) are reported in Figure 3. All quantities are highly correlated with density (*r* > 90%), with *d*_5_, ζ, Ψ, and LSI above 98%. The indicators that less correlate with density (but still >90%) are *V*_4_ and NTC, which also show slightly weaker correlations with the other indicators. This is not really surprising given that they are not explicit descriptors of local structure but rather of energy and “connectivity”. Still, the extremely high values of the correlation coefficients tell us that all these distinct structural properties are strongly coupled: any variation in system density is accompanied by a perfectly concerted response of only apparently distinct structural features. We note in particular that *d*_5_, LSI, and ζ are intercorrelated at *r* ≥ 99.8%.

**Figure 4:**
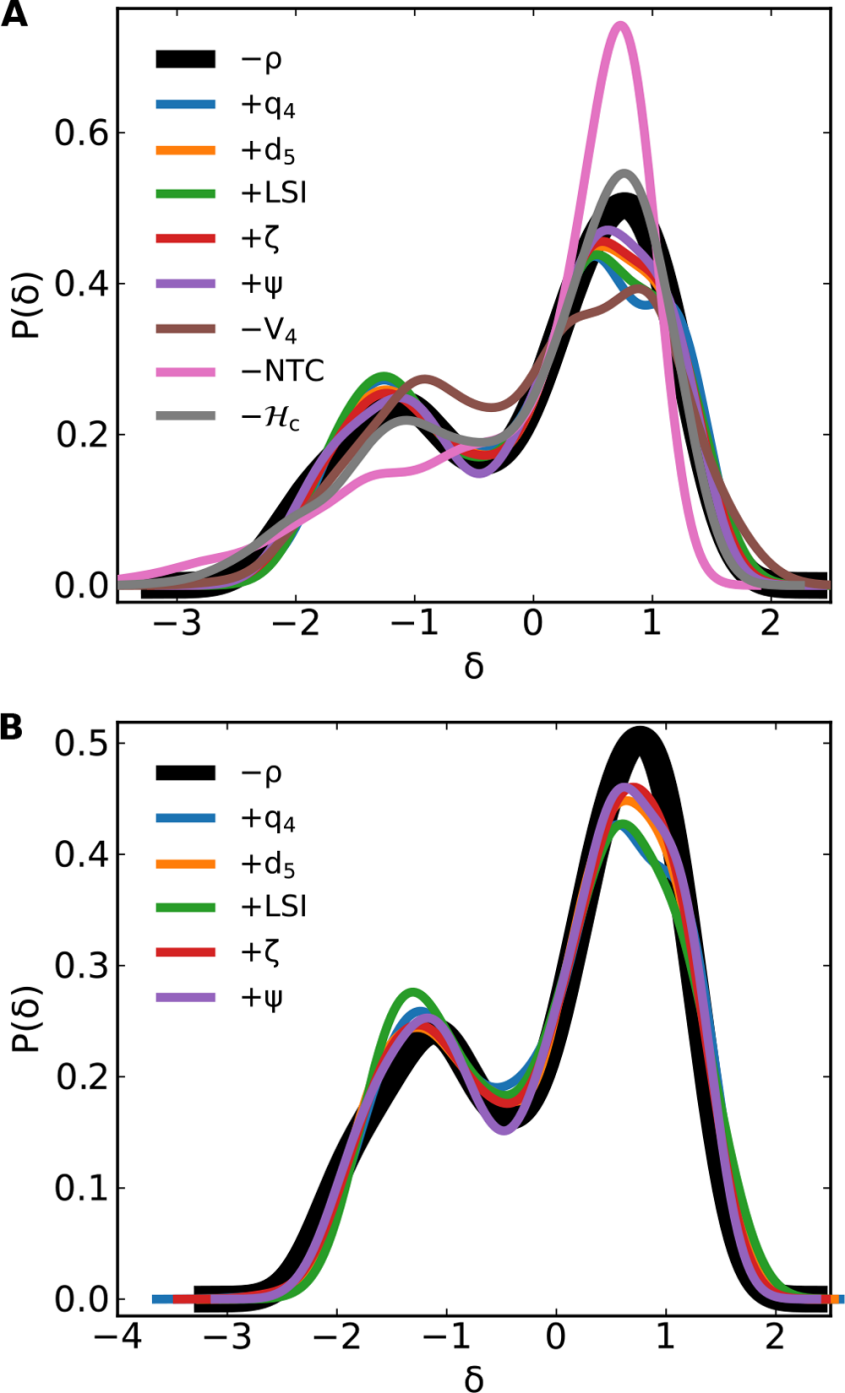
Distribution of density (black, thick) and system-level structural fluctuations (eq 1, color-coded) close to the critical point in TIP4P/Ice water, *T* = 188 K, *P* = 1675 bar, *N* = 300. Curves obtained from kernel density estimations^66^ from data calculated in the inherent structure configurations (A) and in the real dynamics (B, only for some of the indicators). All the quantities that showed global bimodal distributions in the inherent structures (Figure 4) retain the bimodality also in the real dynamics. Except for NTC and *V*_4_, all quantities are described by the same distribution.

Figure 4A shows the distribution of fluctuations displayed by all investigated global structural indicators. Most of them almost exactly match the distribution of density fluctuations. The exceptions are NTC and *V*_4_, which are sampled from different distributions (as verified by a Kolmogorov–Smirnov test^67^ at 95% confidence, with *p* = 0.048 for *V*_4_ and *p* < 10^–9^ for NTC). Since the (logarithm of the) probability distribution in any observable is related to the system free energy, expressed as a function of the same observable, then Figure 4A confirms that all the analyzed indicators, except NTC and *V*_4_, represent essentially the same free energy profile. In other words, they provide—close to the LLCP—the exact same thermodynamic description of the system as the density. All the structural changes that are picked by the different structural indicators are so strongly coupled among themselves (and with density) that, thermodynamically speaking, each of them could be equally well selected as order parameter for the LL transition. At the same time, these results suggest that, close to the LLCP, structural indicators do not provide additional information as compared to density.

We also verified that the same conclusions can be reached by performing the analysis in the real dynamics, instead of the inherent structures. Indeed, the global indicators which displayed a bimodal distribution of fluctuations in the inherent structures also retain this bimodality in the real dynamics (Figure 4B). This is consistent with the fact that the process of energy minimization only removes thermal noise from the system, without affecting the underlying free energy profile.

We highlight that a large correlation coefficient does not per-se imply that the distributions of the fluctuations are identical. Consider the case of NTC. Figure 2G shows that while the density jumps from LDL to HDL are rather well followed by δNTC, the amplitude of the fluctuations inside the LDL and the HDL basins are not properly reproduced. In the LDL basin (high δ) the fluctuations are less intense, resulting in a higher peak in the distribution, while in the HDL fluctuations inside the basin are amplified compared to those of ρ, resulting in large fluctuations that are too spread-out to produce a peak (i.e., same-density configurations can result in different NTC values, implying a sensitivity to some property which is distinct from ρ). In summary, *q*_4_, *d*_5_, LSI, ζ, Ψ, and  not only provide an accurate estimate of the flipping between LDL and HDL configurations but also properly model the fluctuations inside each of these two basins.

**Figure 5:**
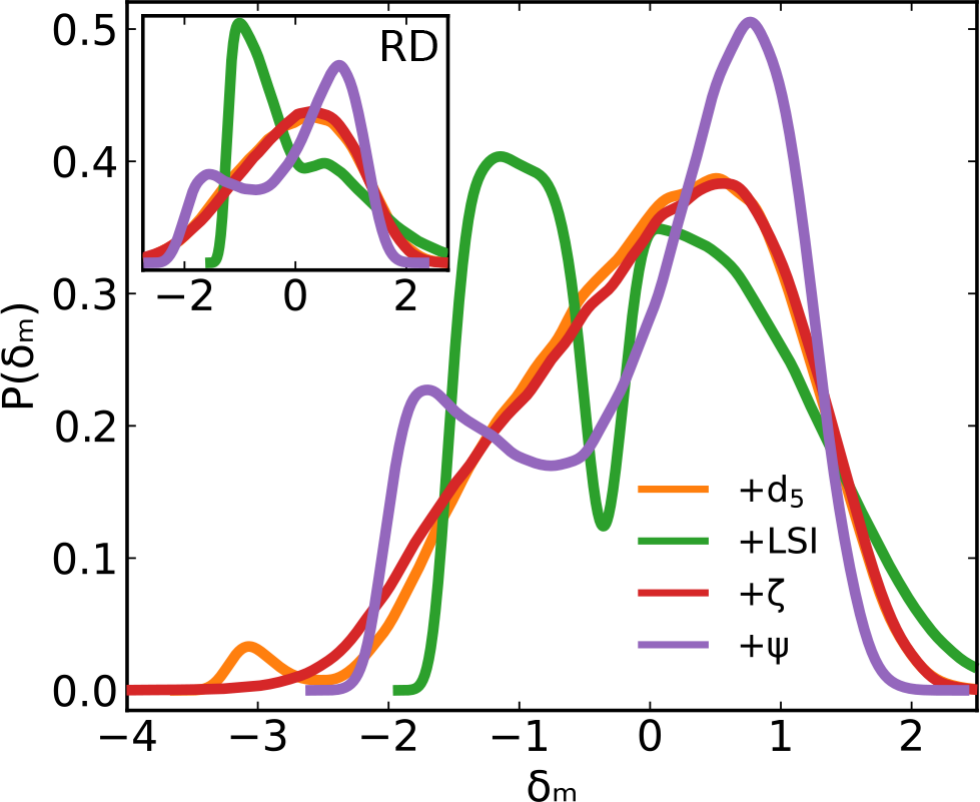
Zero-average and unit variance distribution of structural indicators evaluated at the molecular level (δ_*m*_, eq 3) in the inherent structure configurations. Inset: same distributions evaluated from configurations in the real dynamics. Only LSI and Ψ show molecular-level bimodality, and only Ψ retains this bimodality also in the real dynamics. Data from TIP4P/Ice simulations at *T* = 188 K, *P* = 1675 bar, and *N* = 300 molecules.

We have observed that system-averaged indicators  can detect the transition and some of them even reproduce the correct free energy profile; it is now interesting to investigate whether the transition can also be detected by the molecular-level indicators, . For each molecule *m* we evaluate its fluctuation (dimensionless, with zero mean and unit variance) as
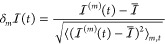
3where  and ⟨ · ⟩_*m*,*t*_ is an average over all molecules *m* in the system and all times *t*. The values of  for each molecule *m* at each time *t* are then pooled together to obtain the distribution of molecular-level structural fluctuations, shown in Figure 5 for the four indicators *d*_5_, LSI, ζ, and Ψ. Despite the proximity of the critical point and the sampling of both LDL and HDL configurations, here ζ and *d*_5_ display (almost identical!) broad unimodal distributions. LSI and Ψ show, instead, clear bimodality, indicating the apparent ability of these two indicators to discriminate quite efficiently the two different local environments. The result for LSI is consistent with previous observations.^68−70^ Interestingly, Ψ (and only Ψ) retains its distinct bimodal character even when configurations from the real dynamics (as opposed to the inherent structures) are used to evaluate δ_*m*_ (Figure 5 inset). In the real dynamics, LSI still shows two peaks but they are not as well-separated as in the inherent structures, a known result that called attention to the possibility that energy minimization could lead to improper characterization of local structures.^47^ The fact that the indicator Ψ, which includes information from four successive HBs (which can span distances from 3 up to 7 Å), retains its bimodality also in the real dynamics, suggests instead that thermal-noise-resilient indicators require the selection of structural properties that sample an extended local region, and that energy minimization is not fundamentally affecting the structure of the liquid.

In studying the LSI distribution we found that its shape (especially at molecular level) is strongly dependent on the choice of the threshold distance, which is used in its definition (as already partially observed by Accordino et al.^71^). The bimodality of the global indicator in the inherent structures is preserved for several threshold distance values, but the area under each peak (and hence the fraction of molecules in low- and high-density local configuration) is strongly threshold-dependent. The extreme sensitivity to this threshold at the single-molecule level calls for additional care when using LSI to perform local structural analysis (see further discussion in the Supporting Information, Figure S1).

**Figure 6:**
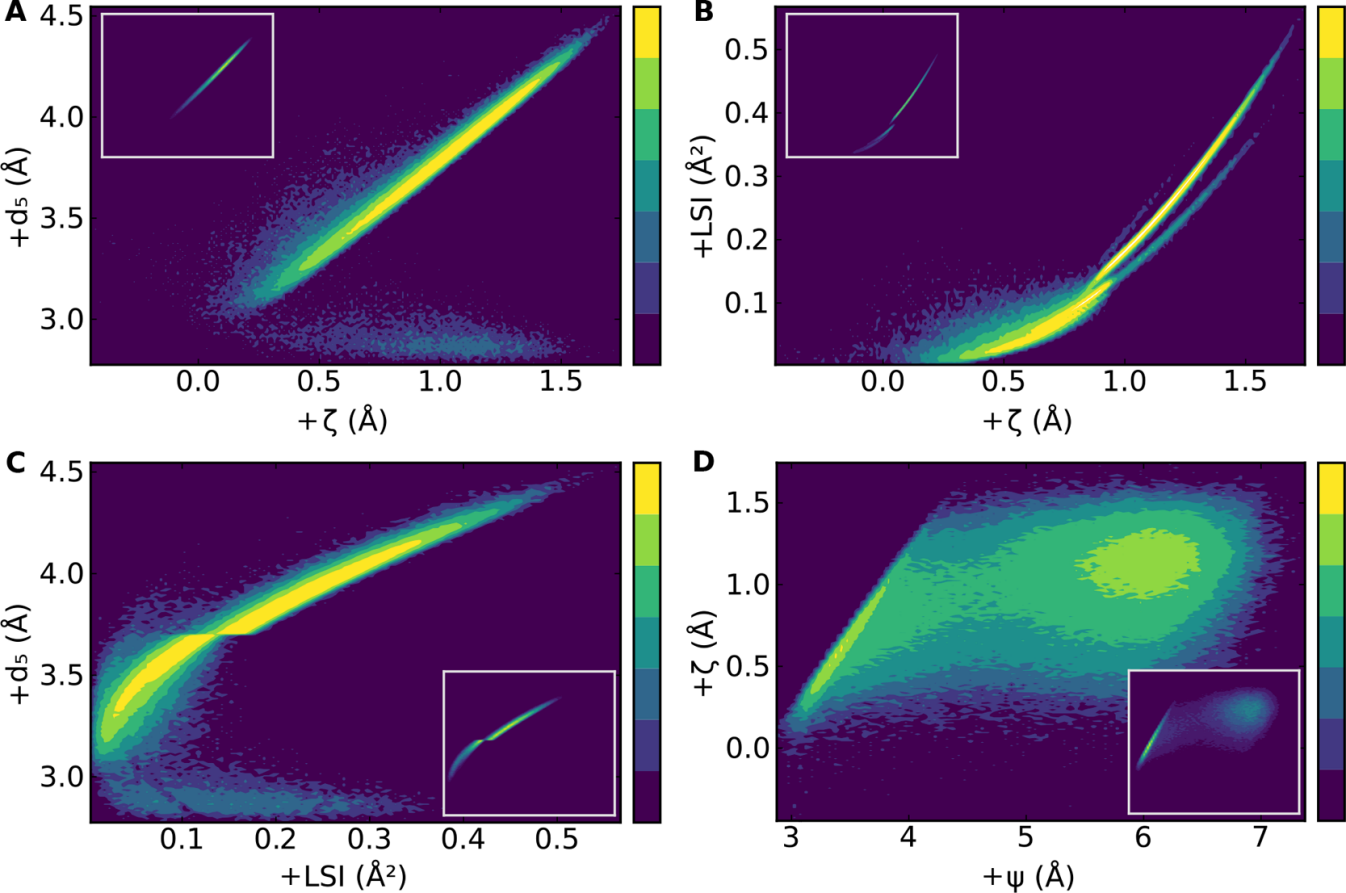
Joint probability distributions for selected pairs of structural indicators in TIP4P/Ice close to the critical point. For each molecule, *d*_5_, LSI, and ζ are in a nearly perfect one-to-one relationship to each other (A–C); other indicator pairs, like ψ–ζ (D) do not have this property. Distributions are obtained by collecting together the indicator values for each molecule in the system at each time step. Main panels show the distribution in logarithmic scale to accentuate features, with each color level corresponding to an order of magnitude increase in relative frequency. Insets show the same distributions in linear scale. Data are from TIP4P/Ice simulations at *T* = 188 K, *P* = 1675 bar, and *N* = 300 molecules.

Molecular level correlations between different pairs of indicators can be analyzed by means of their joint probability distributions (Figure 6); a one-to-one correspondence between two indicators would be represented by a clean monotonic relationship. This is the case for ζ and *d*_5_ (Figure 6A) displaying an almost perfect linear correlation, apart from the very few points with *d*_5_ ≲ 3 Å, signaling the presence of a few molecules with five HBs in LDL-like environments. A strong correlation is also observed between ζ and LSI (Figure 6B) and between LSI and *d*_5_ (Figure 6C) (with the same caveat for the *d*_5_ ≲ 3 Å molecules). The sharp discontinuity in Figure 6C around *d*_5_ ≈ 3.7 Å is, again, an artifact from the threshold used in the evaluation of LSI (which is exactly 3.7 Å in the canonical definition); this effect is discussed in the Supporting Information, where we also show that the location of this discontinuity always corresponds to the threshold in the LSI definition (Figure S2).

Looking at Ψ vs ζ (Figure 6D) we discover, instead, two clearly different basins, one 0.5 Å ≲ ζ ≲ 1 Å and Ψ ≲ 4 Å, and one for ζ ≳ 1 and Ψ ≳ 5 Å; the existence of two distinct basins suggests that these two indicators are not tightly correlated at the molecular level. The bimodality of Ψ highlights the underlying bimodal structure of ζ, which is “masked” by the distance between the two basins being small with the respect to their width.

**Figure 7:**
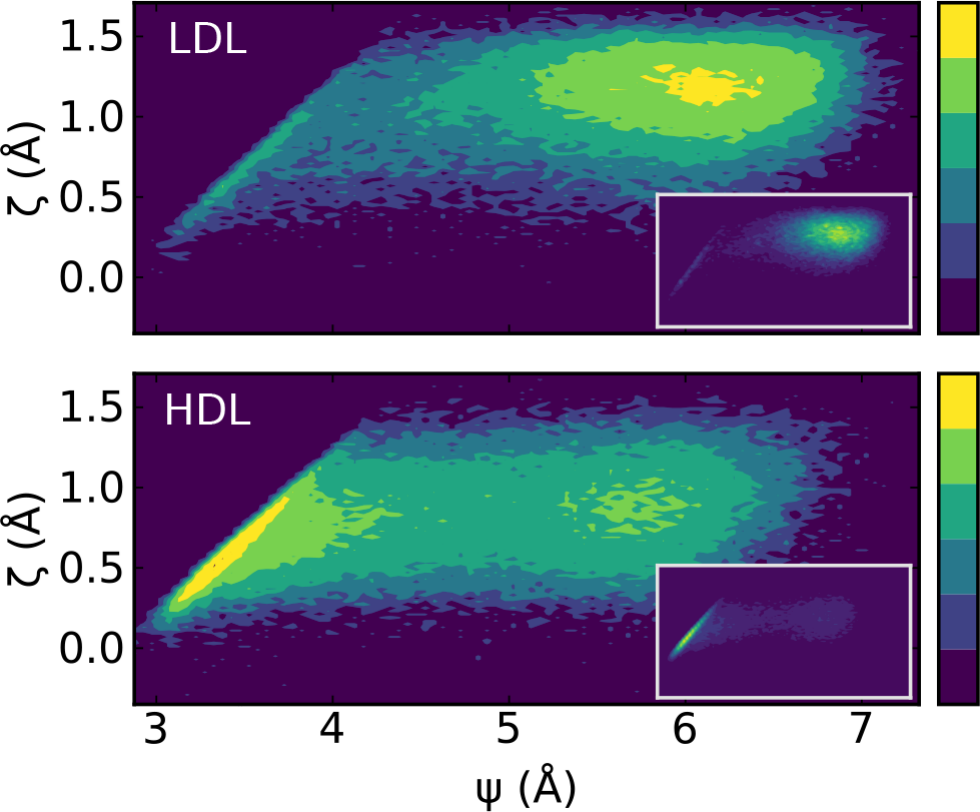
Correlation map between ζ and ψ in LDL and HDL close to coexistence: logarithmic colormap in main panel with each level corresponding to an order of magnitude increase in relative frequency, linear colormap in inset. Data are from TIP4P/Ice simulations at *T* = 188 K, *P* = 1800 bar, and *N* = 1000 molecules (from ref (52)). Due to the proximity to coexistence, the simulation started in the low-density (high-density) liquid phase never crosses to the high (low) phase during the numerical study.

To provide evidence that the two basins in Figure 6D are indeed associated with the LDL and HDL configuration, we show in Figure 7 the corresponding quantities evaluated in both LDL and HDL close to coexistence at *P* = 1800 bar and *T* = 188 K (in a system with 1000 molecules). In this case, critical fluctuations are missing and each trajectory samples only one of the two basins. As expected, the LDL liquid has a strong peak at Ψ ≈ 6 Å at ζ ≳ 1 Å, while the HDL liquid has a strong peak at the interstitial distance Ψ ≈ 3.5 Å and ζ ≈ 0.5 Å.

**Figure 8:**
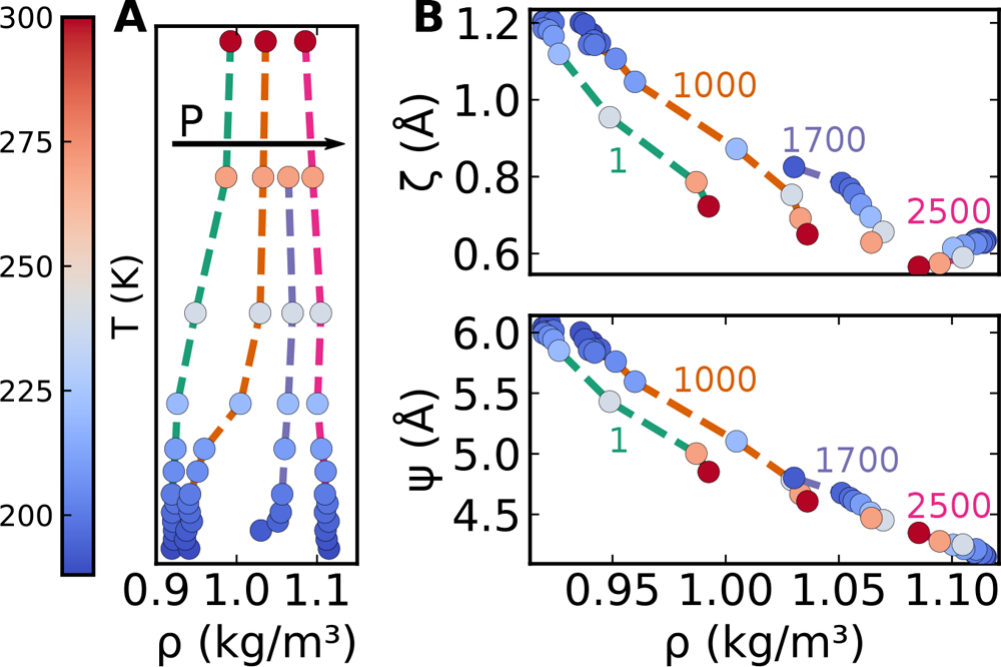
Structural indicators away from the LLCP are not in a one-to-one relationship with density. (A) Four isobars (1, 1000, 1700, and 2500 bar from left to right) in the *T*–ρ phase diagram. (B) Evolution of structural indicators ζ and ψ as a function of density along the four isobars shows a residual dependency, unique for each indicator, on temperature and pressure. In all panels isobars follow the same color-coding and marker colors represent temperature according to the colorbar on the left.

Finally we explore if the observed correlations close to the critical point survive also far from it, when the noncritical part of the free-energy plays a dominant role. Figure 8A shows the density–temperature relationship for four different isobars. At ambient pressure density decreases on cooling, and at 2500 bar density slightly increases on cooling. Figure 8B shows instead the density dependence of the time-averaged value of global indicators  (selecting ζ and Ψ as examples) as a function of the average density. If a state-point-independent correlation existed, all points would lie on the same curve. The observation of different functional forms suggests that away from the LLCP, the identity between global structural indicators () and density (and between different structural indicators) is lost; along the different isobars, there is not a one-to-one correspondence between ρ and the indicators. The ζ–ρ relationship has significant nonlinearities. In particular, along the 2500 bar isobar, the dependency of ζ on ρ is inverted: at that pressure, ρ is slightly decreasing with increasing *T*; ζ, which at lower pressures is negatively correlated to ρ, becomes now positively correlated to it, showing itself a slight decrease (and hence a decrease in order). Interestingly, we note that the relation Ψ–ρ is instead much more linear and does not show the same inversion observed in ζ–ρ: even at high *P*, Ψ is always negatively correlated to ρ, suggesting that the correlation between Ψ and ρ holds in a large phase-space region.

## Conclusions

In thermodynamics and classical theories of liquid state, the relevant order parameter to describe the gas–liquid transition in single-component fluids is density (ρ).^72^ The LLT belongs, like the gas–liquid transition, to the universality class of the 3D Ising model,^73^ so it must be described by a scalar order parameter. Recent numerical results^24,29,30^ provide support to the hypothesis that even in the liquid–liquid transition, density (augmented by an energy correction, the so-called field mixing effect^74^) plays the same role as magnetization in the Ising model. As a caveat, we must note that it is possible to design a model where phases with the same density but different fractions of local motifs coexist; in these ad hoc cases ρ cannot be used as an order parameter for the liquid–liquid transition.^41^

There have been, however, also proposals for a two-order-parameter theory of water,^75−77^ expressing the liquid free energy in terms of ρ and of an additional scalar nonconserved parameter, a structural indicator. It has been also argued that the density is the order parameter relevant for the gas–liquid transition, whereas a structural order parameter is relevant for the liquid–liquid transition.^77^ In this view, the difference in density between HDL and LDL is a consequence of the intrinsic density difference of the two local structures.^77^

Here, analyzing molecular dynamics simulations of the TIP4P/Ice water model close to its liquid–liquid critical point, we compared critical density fluctuations to the structural fluctuations as described by different structural indicators (*q*_4_, *d*_5_, LSI, ζ, *V*_4_, NTC, and a newly defined Ψ). Our analysis shows that all these indicators are capable of detecting, with high accuracy, transitions between the two liquid states, showing a near-perfect correlation to density and also to other indicators (Figures 2 and 3). Furthermore, we observed that most of them display the same distribution of fluctuations as the density, implying that they probe the same free energy landscape (Figure 4). Therefore, close to the critical point, all these indicators are identical to the density and describe the exact same thermodynamic behavior. In this respect, the liquid–liquid transition of water can be equally well described as a transition driven by density or a transition driven by a change in the structural properties.

Since in proximity of the LLCP most of the indicators behave exactly the same as density, to understand the LLT of supercooled water from a thermodynamic viewpoint there is in principle no necessity for a two-order-parameter description of the free energy. Concurrently, there is no loss in information nor accuracy in using such order parameters as an alternative to density, if density is expressed as a linear combination of the fraction of molecules of each type. Moving away from the critical point, the noncritical standard component of the free energy becomes dominant. Correlations between density and structural indicators progressively degrade (but less for Ψ), and specifying the density alone is not sufficient anymore to completely describe the structure of the liquid.

We have also shown that the newly proposed indicator Ψ outperforms all other structural indicators in its ability to identify the local environment of each molecule (Figure 5). While in the low-density liquid, molecules at chemical distance *D* = 4 (distance measured in units of hydrogen bonds) are quite far (relative distance about 6–6.5 Å), in the HDL, the hydrogen bond network folds in, bringing the *D* = 4 molecule into the first shell of the selected molecule (relative distance about 3–3.5 Å).

A study related to our work^78^ has appeared during the review process, focusing on some of the indicators discussed herein, as well as on additional structural parameters based on the Delaunay tesselation. They observe that, besides LSI, also the volume and aspect ratio of Delaunay tetrahedra express bimodality close to the critical point, but only with a weak correlation to the system density.

The study of structural indicators has provided great insight into the microscopic behavior of water, and with more and more descriptors becoming available in recent years, our understanding of the local structures of the two liquid states is continuously increasing. We have now a clear picture of the tight coupling that the critical phenomenon enforces between many, apparently distinct, structural properties, where a small fluctuation in any one of these, will be accompanied by a concerted response in all of the others. It is thus foreseeable that several of these indicators can be chosen to numerically investigate nucleation of one liquid into the other with a global order parameter; but studies based on a molecular description (more appropriate to investigate nucleation) should possibly rely on indicators showing unambiguous molecular-level bimodality, such as Ψ. The evidence of a tight coupling between several different structural properties close to the critical point requires a further effort to identify and visualize the structural changes that simultaneously require an increase in density, node total communicability, and *V*_4_ and a decrease in the distance of the fifth neighbor *d*_5_, in the LSI, in tetrahedrality (*q*_4_), in ζ, Ψ, and , and so on.

## Data Availability

The data that support the findings of this study are available within the article. Additional data (MD trajectories) are available from the corresponding author upon reasonable request.

## Supporting Information Available

Description of the new indicator Ψ, analysis of the behavior of LSI (PDF)
