# Supplementary material for: Correlated Fluctuations of Structural Indicators Close to the Liquid–Liquid Transition in Supercooled Water
Source: J Phys Chem B. 2022 Dec 20;127(1):378–86. doi: 10.1021/acs.jpcb.2c07169 (PMC9841516; doi:10.1021/acs.jpcb.2c07169)
Supplement: Supplementary file 1 — jp2c07169_si_001.pdf [file jp2c07169_si_001.pdf]

Supporting Information: Correlated Fluctuations of Structural Indicators Close to The Liquid-Liquid Transition in Supercooled Water

Riccardo Foffi[†] and Francesco Sciortino[*,‡]

†*Institute for Environmental Engineering, Department of Civil, Environmental and Geomatic Engineering, ETH Zürich, Zürich, Switzerland.*

‡*Dipartimento di Fisica, Sapienza Università di Roma, Piazzale Aldo Moro 5, I-00185 Rome, Italy.*

E-mail: francesco.sciortino@uniroma1.it

The indicator Ψ

The novel indicator Ψ introduced in the text is based on topological properties of the HB network of water, following observations from Ref.[S1,S2] After mapping the HB network using the HB definition of Luzar and Chandler[S3], we associate to each pair of molecules in the system a chemical distance D, which is the length (measured in number of HBs) of the shortest path between two molecules along the network. The set of molecules at a given chemical distance D from a reference molecule i, constitutes the D-th bond-coordination shell of i. It was observed[S1,S2] that the transition from the LDL to HDL state is associated to an interpenetration phenomenon between the 2nd and 4th bond-coordination shells, becoming more and more pronounced as density is increased: in the HDL, the HB network folds in and

pairs of molecules at $D = 4$ are found at spatial distances much shorter than in the LDL, where the tetrahedral arrangement enforces large spatial distances between topologically distant molecules. For any given molecule, we identify all the molecules in its 4th bond-coordination shell (i.e. at chemical distance $D = 4$), and define the value of Ψ for that molecule as the spatial distance from the closest molecule in said shell. Tetrahedral, low-density configurations will then be characterised by large values of Ψ ($\sim 6.5\,\text{Å}$), while high-density structures favor shorter distances ($\sim 3.5\,\text{Å}$).

On the behavior of LSI

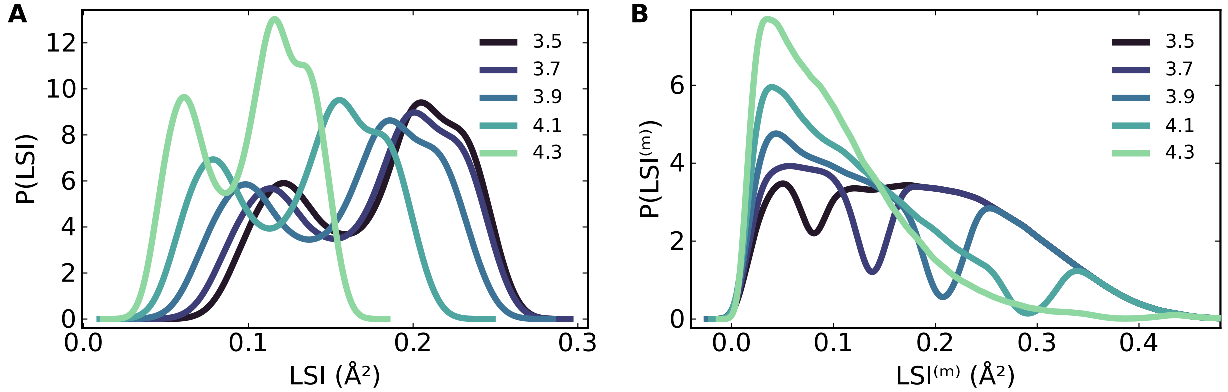

Figure S1: The choice of the threshold radius in the definition of LSI (Equation 1) affects its distribution. (A) Distribution of global LSI for varying r_{th} threshold (legend, in Å). Different threshold only result in a shift of the distribution. (B) Distribution of molecular-level LSI$^{(m)}$ for varying r_{th} thresholds. Here the choice of r_{th} affects the shape of the distribution drastically. For both panels, data was extracted from the inherent structures configurations.

In the main text we have shown that, along with Ψ, LSI is the only structural indicator to preserve bimodality at single-molecule level, even if only in the inherent structures (Fig. 5). The observed bimodality of LSI is, however, strictly dependent on the choice of a threshold value in its definition. Nearest-neighbors are ranked by their oxygen-oxygen distance from a central molecule m as $r_1 < r_2 < \ldots < r_i < r_{\text{th}} < r_{i+1}$ where i is chosen so that $r_i < r_{\text{th}} < r_{i+1}$;

then

$$\mathrm{LSI}^{(m)} = \frac{1}{i}\sum_{j=1}^{i}(\Delta(j) - \bar{\Delta}(j))^2 \tag{1}$$

where $\Delta(j) = r_{j+1} - r_j$ and $\bar{\Delta}$ is the average of $\Delta(j)$ over the i neighbors of m. So LSI provides a measure of the fluctuations in the distance distribution surrounding a given water molecule within a sphere of radius r_{th}.

The original definition[S4] and all subsequent works[S5–S10] adopted the threshold $r_{\mathrm{th}} = 3.7\,\text{Å}$, a distance that lies between the first and second neighbor shells. At the global (i.e. system-averaged) level, the choice of a different threshold results mainly in a shift of the distribution, without qualitatively altering the structural description (Figure S1A). At the molecular level, instead, r_{th} drastically affects the shape of the distribution (Figure S1B). Following the rationale that a proper choice of r_{th} should lie somewhere in between the first and second coordination shells, any value in the range $[3.5, 3.9]$Å could be an equally reasonable choice. While this would have no effect on the bimodality of the global indicator, and therefore on its ability to describe the thermodynamics of water close to the LLCP, the local structural picture provided by LSI would be extremely sensitive to this choice; therefore, care must be taken when using LSI for local structural analysis.

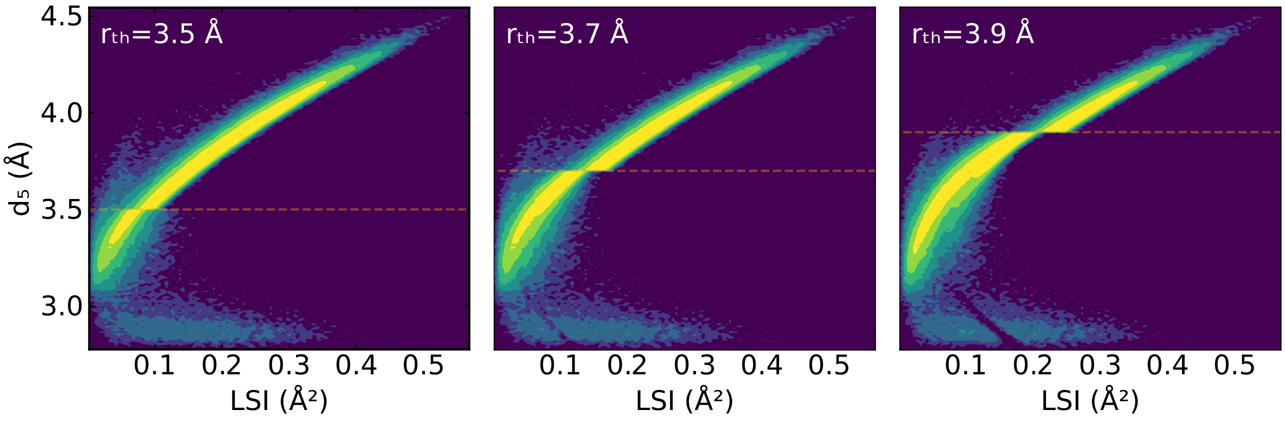

Figure S2: Joint probability distributions for d_5 and LSI, with varying thresholds r_{th} for LSI. The location of the discontinuity coincides with the threshold r_{th} used in the definition of LSI. All molecules whose fifth neighbor is farther than r_{th} have that neighbor excluded from the LSI evaluation.

Figure S2 clearly shows, as mentioned regarding Fig. 6C, that the existence of a discontinuity in the d_5–LSI joint distribution, created by the selected r_{th} value, is the source of bimodality in the molecular distribution. This is apparent considering that distribution of LSI is the projection of the d_5–LSI joint distribution on the LSI axis. Each of the two branches of the d_5–LSI joint distribution produces its own peak in the LSI distribution. The minimum that appears in the LSI distribution is thus induced by the value of r_{th}. The low-LSI peak reflects the molecules with more than four neighbours within r_{th}, the high-LSI peak those less than five. In fact, for each molecule whose fifth neighbor is farther than r_{th} (i.e. $d_5 > r_{\mathrm{th}}$), this neighbor is excluded from the evaluation of LSI. This is a further point that calls for attention when using LSI to obtain information about the local structure of single molecules and to quantify the bimodal character of the local environment.[S5,S7]

References

(S1) Foffi, R.; Russo, J.; Sciortino, F. *J. Chem. Phys.* **2021**, *154*, 184506.

(S2) Foffi, R.; Sciortino, F. *Phys. Rev. Lett.* **2021**, *127*, 175502.

(S3) Luzar, A.; Chandler, D. *Nature* **1996**, *379*, 55–57.

(S4) Shiratani, E.; Sasai, M. *J. Chem. Phys.* **1996**, *104*, 7671–7680.

(S5) Appignanesi, G. A.; Rodriguez Fris, J. A.; Sciortino, F. *Eur. Phys. J. E* **2009**, *29*, 305–310.

(S6) Gelman Constantin, J.; Rodriguez Fris, A.; Appignanesi, G.; Carignano, M.; Szleifer, I.; Corti, H. *Eur. Phys. J. E* **2011**, *34*, 1–5.

(S7) Wikfeldt, K. T.; Nilsson, A.; Pettersson, L. G. M. *Phys. Chem. Chem. Phys.* **2011**, *13*, 19918.

(S8) Shi, R.; Tanaka, H. *J. Chem. Phys.* **2018**, *148*, 124503.

(S9) Martelli, F. *J. Chem. Phys.* **2019**, *150*, 094506.

(S10) Tanaka, H.; Tong, H.; Shi, R.; Russo, J. *Nat. Rev. Phys.* **2019**, *1*, 333–348.
